# Multimodality Image Analysis in a Cohort of Patients with Atypical Juvenile Ocular Toxocariasis

**DOI:** 10.1155/2021/4853531

**Published:** 2021-02-11

**Authors:** Xiaohong Guo, Hui Liu, Manli Li, Ke Fan, Shuyin Li, Bo Lei

**Affiliations:** Henan Provincial People's Hospital, Henan Eye Institute, Henan Eye Hospital, People's Hospital of Zhengzhou University, Zhengzhou, Henan 450003, China

## Abstract

**Purpose:**

To analysis the multimodal imaging of a group of patients diagnosed clinically with atypical juvenile ocular toxocariasis (OT).

**Methods:**

In this case series study, we examined 9 young patients diagnosed with atypical OT. Routine ophthalmological examinations, fundus photography, optical-coherence tomography (OCT), fluorescein angiography (FFA), and B-mode ultrasound were performed. A questionnaire was used to record whether the patients were newly diagnosed and whether they had a history of exposure to a cat and dog. Aqueous humor and serum samples were taken for serological tests.

**Results:**

In all the patients, yellow-and-white dot-shaped lesions and perivascular white sheath were seen in the fundus. Heterogeneous changes including hyper-reflection in the disrupted neuroretina, hyper-reflection in the outer retinal layer, high-reflection mass on the surface of the neuroretina accompanied with reflective attenuation, and high-reflection mass involving the entire neuroretina or high-reflection mass in the vitreous body were noticed in OCT images. On FFA, seven of these patients (77.8%) showed leakage of fluorescein in the small- and medium-branch veins of the retina, and a “bristle-like” change indicated increased permeability of the vessels. B-mode ultrasound showed proliferative membranes and proliferative bands (33.3%), as well as spotted opacity in the vitreous (66.7%). The antibodies to *Toxocara canis* in the aqueous humor and serum were positive, and the Goldmann–Witmer coefficient was significantly increased in 6 out of 7 patients.

**Conclusions:**

Multimodality images are useful in the diagnosis of atypical juvenile OT, which could be easily overlooked and misdiagnosed.

## 1. Introduction

Toxocariasis is a parasitic disease caused by *Toxocara canis* or *T. cati* larvae worldwide. Eggs ingested by humans hatch into parasitic larvae in the small intestine. Through blood circulation, larvae may invade the eyes and cause ocular toxocariasis (OT). OT usually is unilateral and commonly affects adolescent patients in the European, American, and Chinese populations. Due to the involvement of many factors including the site of invasion and the course of the disease, the clinical manifestations of OT are heterogeneous, which makes a correct diagnosis difficult. Although biopsy is the most valuable method for the diagnosis of OT [1], aqueous humor and blood serological tests, together with a history of pet exposure, are also among diagnostic criteria. Unfortunately, some relevant data usually are not always available from all patients. Therefore, the importance of careful observation of the ocular manifestations for the diagnosis of OT is exaggerated.

According to its manifestations, OT can be divided into four types: peripheral granuloma, posterior polar granuloma, chronic endophthalmitis, and mixed type. Although typical OT can be diagnosed by signs of retinal granulomatosis or toxocariasis endophthalmitis [2], OT patients with atypical manifestations are likely to be misdiagnosed. These atypical OTs include involvement of the ciliary-body [3] or lens [4] invasion, intraocular tumors, and optic-nerve swelling [5]. Recently, improved multimodal ophthalmic-imaging technologies have unveiled the details of retinal lesions in OT. Analysis of multimodal ophthalmic images, combined with medical history and immunological examinations, has markedly improved the diagnosis for this relatively unaware condition. In this study, we performed multimodal-imaging analysis in a cohort of patients diagnosed clinically with atypical juvenile OT and explored the characteristics of the imaging.

## 2. Patients and Methods

Nine OT patients diagnosed clinically with atypical granulomas in our hospital from January 2016 to December 2019 were included. The aqueous humor was collected, and an enzyme-linked immunosorbent assay (ELISA) was performed to detect *Toxocara* antibodies. The Goldmann–Witmer coefficient (GWC), which was calculated as ([specific IgG in aqueous humor/specific IgG in serum]/[total IgG in aqueous humor/total IgG in serum]), was obtained [6]. The diagnosis of OT was made when the specific *Toxocara* IgG in the aqueous humor was positive. Patients with granuloma that could be caused by other conditions including toxoplasmosis, sarcoidosis, uveitis, and fungal infections were excluded. Patients with a history of ocular trauma or surgery were also excluded. The research procedure was approved by the Institutional Ethics Committee of the Henan Eye Hospital. Guardians of all patients signed an informed consent form.

Best corrected visual acuity (BCVA), slit lamp, and direct ophthalmoscopic results of all patients were obtained. A questionnaire was used to record whether patients were newly diagnosed and whether they had a history of exposure to a cat and dog. An Optos Optomap Panoramic 200 laser scanning ophthalmoscope (OptosPLC, Dunermline, UK) was used to collect color photographs of the fundus with a range of 200°. We used either spectral-domain or swept-source optical-coherence tomography (Heidelberg SD-OCT, Germany and VG200D SVision Imaging SS-OCT, China) for scanning the retina. Fluorescein angiography (FFA) was performed with a Heidelberg confocal laser scanning fluorescein angiography apparatus (Heidelberg Engineering GmbH, Heidelberg, Germany). An ultrasound (MD-2400S MEDA, China) was used to observe the vitreous bodies.

## 3. Results

Nine patients with OT were included in this study (6 males and 3 females; aged from 6 to 16 years, average 10.11 years). All the patients presented unilateral OT. The BCVA range was 0.01–0.8, with an average visual acuity of 0.31. Slit-lamp examination showed flare (+) and cells (+) in the anterior chamber in 5 patients (55.6%), while the remaining 4 (44.4%) did not exhibit noticeable abnormalities in the anterior segment. Fundus examination revealed no typical peripheral or posterior-pole granulomas. Six patients (66.7%) had histories of exposure to a cat or dog, while the other 3 (33.3%) did not. Immunological examinations of both serum and the aqueous humor were performed in 7 patients (77.8%), and only of those of the aqueous humor were conducted in the other 2 patients (22.2%). Details of all 9 patients are shown in Table 1.

**Figure 1:**
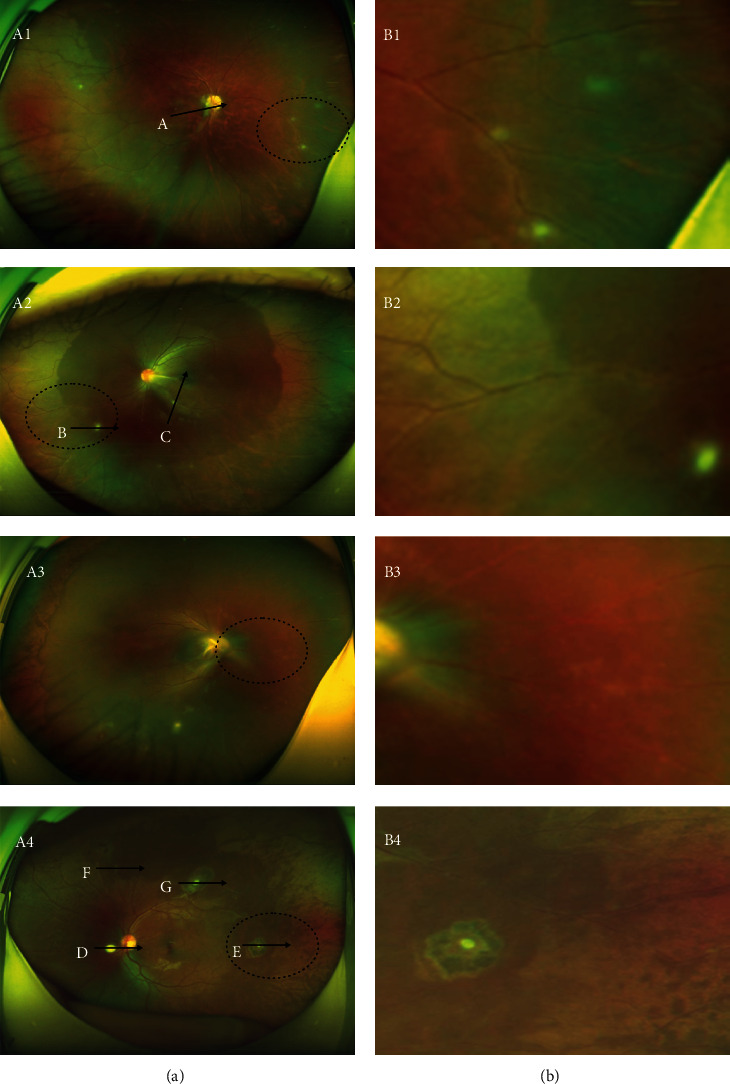
Panoramic fundus photos: yellow-and-white dot-shaped lesions were visible. Enlarged fundus photos show the perivascular white sheath (oval dotted lines indicate the selected area corresponding to figure (B1), (B2), (B3), and (B4), and A, B, C, D, E, F, and G scan lines correspond to Figure 2).

**Figure 2:**
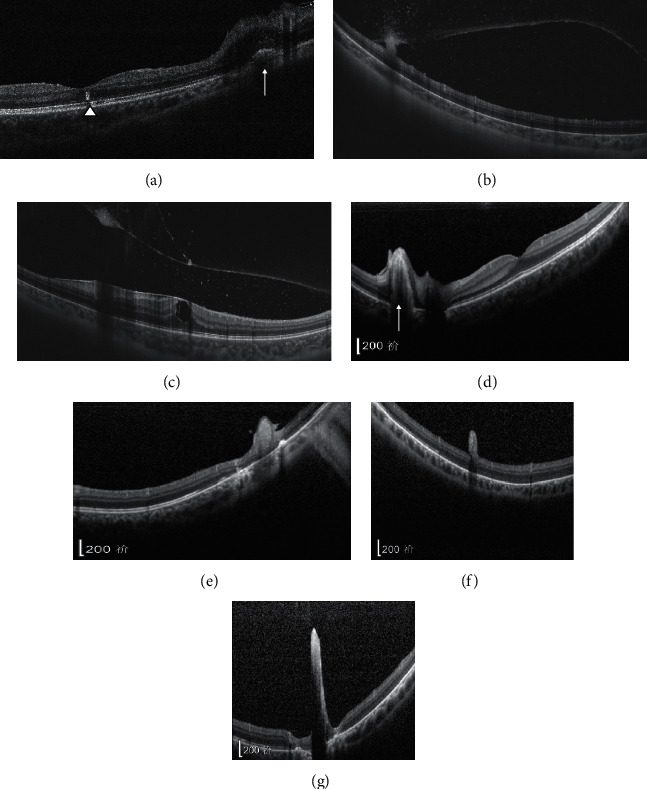
OCT images of yellow-and-white punctate lesions marked in Figure 1. The lesions were heterogeneous hyper-reflective in the neuroretina (a (arrow) and d), hyper-reflective in the outer retina (a (triangle)), high-reflective mass on the surface of the neuroretina with a reflective attenuation beneath (b and f), high-reflective mass involving the entire layer of the disrupted retina (e and g), and high-reflective mass located in the vitreous body (c).

In 8 out of 9 patients (88.9%), yellow-and-white dot-shaped lesions ranging from 80 to 300 *μ*m in diameter were observed in the fundus (Figure 1). In the remaining cases (11.1%), no obvious yellow-and-white dot-shaped lesions were seen. In panoramic fundus photos, a perivascular white sheath was evident (Figure 1, B1 B2 B3 B4). A total of 20 yellow-and-white dot-shaped lesions were seen in eight patients. Ten of these lesions (50%) were able to be scanned by OCT. Among them, 2 (20%) showed heterogeneous hyper-reflection in the disrupted neuroretina (Figure 2(a) arrow and 2(d)) and 2 (20%) showed hyper-reflection in the outer layer of the neuroretina (Figure 2(a) triangle). In 3 (30%) lesions, a high-reflection mass was seen on the surface of the neuroretina (Figures 2(b) and 2(f)). In 2 (20%) lesions, a high-reflection mass involving the entire neuroretina was seen (Figure 2(e) and 2(g)). One lesion (10%) was a high-reflection mass located in the vitreous body (Figure 2(c)).

**Figure 3:**
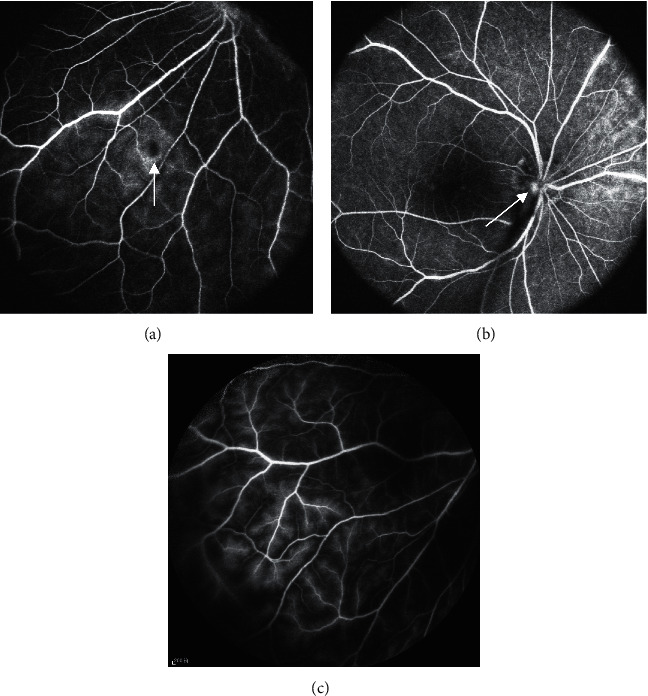
(a) The blocked fluorescence which displayed a yellow-white punctate lesion (arrow) in the fundus picture. (b) The fluorescein leakage in the yellow-white punctate lesion (arrow). (c) The “bristle-like” leakage.

FFA was not performed in one patient due to allergies and in another 6-year-old patient who was uncooperative. In the remaining 7 patients (77.8%), the yellow-and-white fundus lesions observed in fundus pictures showed blocked fluorescence or leakage on FFA. Six of these patients (85.7%) showed “bristle-like” leakage of fluorescein in the small- and medium-branch veins of the retina in the middle and late stages of angiography, suggesting the permeability of the blood vessels was increased. No retinal blood vessel leakage was observed in the remaining patients (11.1%) (Figure 3).

**Figure 4:**
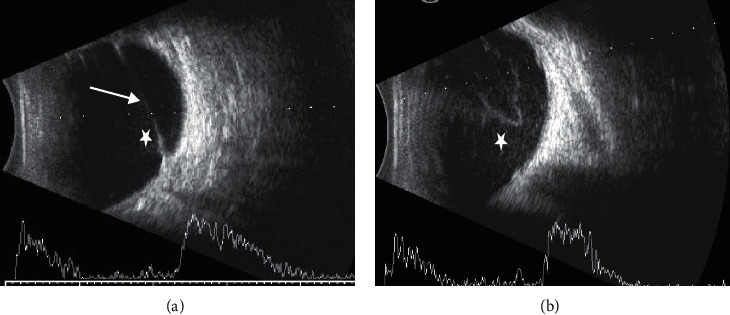
B-mode ultrasound showed vitreous membranes and bands (arrow) and punctate interfaces (stars) usually related to inflammatory changes of the vitreous. Panels a and b were from patient no. 2 and no. 4 in Table 1, respectively.

In B-mode ultrasound, 3 cases (33.3%) exhibited vitreous membranes and bands and 6 cases (66.7%) showed punctate interfaces usually related to inflammatory responses of the vitreous (Figure 4).

## 4. Discussion

Although toxocariasis is the most common helminthic infection of humans in industrialized countries [7], the public is poorly aware of its complications [8]. In addition to the typical granulomatous manifestations, there are also atypical toxocariasis-related fundus manifestations. The variable clinical manifestations make a correct diagnosis of OT difficult, and patients tend to be misdiagnosed in clinical practice.

According to previous reports, the demographic characteristics of OT differ by region. In the European and US populations, most OT patients were children, while in South Korea and Japan, most affected patients were adults, presumably attributed to food habits [9, 10]. In this case series, all 9 patients were young. As young patients may often have ambiguous complaints and atypical manifestations, ocular toxocariasis, especially at earlier stages, could be misdiagnosed.

In this study, four of atypical OT patients (44.4%) were misdiagnosed in other hospitals before visiting our clinic. They had received diagnoses of periphlebitis, uveitis, vasculitis, and/or retinal vasculitis in local hospitals. Consequently, it might have caused delays in treatment or even inappropriate management. Therefore, it is crucial to apply multiple imaging techniques to improve the diagnosis of the disease. Three patients (33.3%) in this study had no histories of exposure to pets, and they might have been exposed to soil contaminated with *Toxocara* eggs. A study found that 13%–87.1% soil samples collected from parks were 13%–87.1% positive for *Toxocara* eggs, especially samples taken in certain rural and urban areas [11, 12]. In this study, 6 atypical OT patients (66.7%) had a BCVA of 0.2 or higher, which might also interfere diagnosis. Because the remaining visual acuity of these children was still useful, the disease itself was likely to be overlooked. Two patients (22.2%) underwent vitrectomy due to vitreous opacities in this study, while no obvious granulomas were seen during the operations. Nevertheless, a diagnosis of OT was made based on immunological detection combined with postoperative-imaging findings. Thus, it is recommended that OT should be considered in patients with unknown causes of uveitis and endophthalmitis.

All patients in this study were clinically diagnosed based on intraocular inflammation, and immunological findings after other ocular infections were excluded. It have been confirmed that specific antibodies exist in the eyes of OT patients. In our study, serum and aqueous humor anti-*Toxocara canis* IgGs were positive in all 7 patients tested, and all patients but 1 (11.1%) displayed a Goldmann–Witmer coefficient greater than 4. Although the GWC of the remaining one patient was less than 4, both serum and aqueous humor Anti-*Toxocara canis* IgG levels were significantly increased in this case, indicating a status of infection.

We applied multimodal fundus imaging to illustrate changes in the fundus associated with OT. In particular, we used high-resolution OCT combined with panoramic fundus photography to describe the lesion. Ultra-wide-angle fundus photos showed scattered yellow-and-white lesions with diameters ranging 80–300 *μ*m located at the posterior pole or near the equator. Unlike the common granuloma of typical OT, these yellow-and-white ocular lesions were small in size (microgranulomatous) [13]. Microgranulomas were located outside or inside the neuroretina on OCT [14]. At the same time, premacular membrane, intraocular proliferative membrane, and vitreous opacity were among the common concomitant symptoms of OT. We showed that the yellow-and-white lesions (microgranulomas) in the eye displayed not only at all layers of the neuroretina but also in the vitreous. Nevertheless, the specific components of the lesion deserve further investigation.

Since the first study by Dernouchamps et al. [15] in 1990, imaging features of OT from FFA had been rarely reported. We identified leakage of the middle and small vein in the retina at the late stages of FFA, indicating the permeability of blood vessels was increased. The corresponding lesion observed in the fundus was the perivascular white sheath. On the contrary to the reports by Dernouchamps et al. [15], most of the increased permeability of small blood vessels that we saw did not involve the macula, but rather the branches of small and medium veins in the periphery area. Interestingly, a “bristle-like” leakage image on angiography was evident. While microgranulomas in the vitreous, in front of the retina, and in the retina blocked the fluorescein, microgranulomas located under the neuroretina mostly exhibited fluorescein leakage on FFA. The leakage may be related to the damage of the outer blood retinal barrier, presumably caused by migration of larvae from the choroid to the retina. It also might be related to retinal and choroidal inflammation associated with the lesion. In one patient, we saw no significant leakage or dilatation of small blood vessels. Possibly, this patient was at a late phases of OT. In younger children, vision loss might be overlooked and the patient might be misdiagnosed. When the symptom was finally noticed and a correct diagnosis was made, the inflammatory response might have been controlled and the permeability of the blood vessel recovered.

Ocular B-mode ultrasound can reflect various vitreoretinopathies in OT, especially the severity of intraocular proliferation and inflammation. Various proliferative membranes and bands in the affected eye made them supplement evidence for OT diagnosis.

## 5. Conclusions

In summary, imaging manifestations of atypical juvenile OT presented the following characteristics: intraocular proliferative membranes and bands, vascular white sheaths, yellow-and-white lesions of the fundus that could affect any layer of the retina, and “bristle-like” leakage of small and medium veins in FFA. For children with such manifestations, the possibility of OT should be considered. Multimodality image analysis combined with immunological tests, as well as a history of pet exposure, should be used for diagnosis.

## Figures and Tables

**Table 1:** The clinical data and immunological detection results of 9 patients with OT.

Case	Gender	Age (year)	Newly diagnosed patient: yes or no	Contacted with dogs or cats: yes or no	Affected eye	BCVA	Slit-lamp examination	Aqueous humor anti-*Toxocara canis* IgG (U/L)	Serum anti-*Toxocara canis* IgG (U/L)	GWC
1	Male	7	Yes	Yes	OS	0.8	No	5.45	15.08	31.41
2	Female	14	No (diagnosed as periphlebitis)	Yes	OD	0.3	Anterior chamber flare (+) cells (+)	6.68	5.96	194.19
3	Male	8	Yes	Yes	OD	0.15	Anterior chamber flare (+)	31.72	39.89	21.7
4	Male	10	Yes	Yes	OS	0.07	No	4.92	5.94	70.18
5	Female	12	Yes	Yes	OD	0.8	Anterior chamber flare (+) cells (+)	7.04	11.9	102.9
6	Male	7	No (diagnosed as fundus hemorrhage)	No	OS	0.2	No	27.91	7.64	97.84
7	Male	6	Yes	Yes	OS	0.01	No	50.43	41.61	2.39
8	Male	11	No (diagnosed as uveitis)	No	OS	0.25	Anterior chamber flare (+) cells (+)	31.18		
9	Female	16	No (diagnosed as retinal vasculitis)	No	OS	0.2	Anterior chamber flare (+) cells (+)	37.89		

Note: patient no. 6 and 7 underwent vitrectomy because of vitreous opacity, no obvious granuloma was found during the operation, and the imaging examinations (except B-mode ultrasound) were the results of postoperative examination. Patient no. 8 and 9 performed only an aqueous humor test for *Ascaris lumbricoides* IgG. GWC: Goldmann–Witmer coefficient. The GWC was calculated as ([specific IgG in aqueous humor/specific IgG in serum]/[total IgG in aqueous humor/total IgG in serum]).

## Data Availability

Te data used to support the findings of this study are available from the corresponding author upon request.
